# Proximal subscapularis release for the treatment of adduction–internal rotation shoulder contracture in obstetric brachial plexus palsy

**DOI:** 10.1007/s11832-015-0696-2

**Published:** 2015-09-30

**Authors:** Elias Naoum, Elie Saghbini, Elias Melhem, Ismat Ghanem

**Affiliations:** Department of Orthopaedic Surgery, Hotel-Dieu de France Hospital, Saint Joseph University, Boulevard Alfred Naccache, Achrafieh, P.O. Box: 166830, Beirut, Lebanon

**Keywords:** Brachial plexus palsy, Erb’s palsy, Adduction–internal rotation contracture, Subscapularis release

## Abstract

**Introduction:**

The purpose of this paper was to evaluate the results on shoulder function following isolated proximal subscapularis release in children with Erb’s palsy.

**Methods:**

A retrospective study was conducted on 64 consecutive children with Erb’s palsy who underwent a Carlioz proximal subscapularis release between 2001 and 2012. Fifty children with complete records and a minimum follow-up of 2 years were included for evaluation. Age at surgery ranged from 1.3 to 4.5 years (average 2.6 years). Preoperative active shoulder abduction/anterior elevation, active external and internal rotations as well as the Mallet score were compared with those found at 6 and 24 months postoperatively using the Student paired *t* test, with a confidence interval of 95 %. The results were compared between children <3 years of age at surgery and those older, and between children who had an isolated C5–C6 and those with greater involvement. *p* < 0.05 was considered statistically significant.

**Results:**

Active abduction improved 21° at 6 months and 31° (total) at 2 years (*p* < 0.01) with an overall Mallet abduction score improvement of 0.58 at 6 months and 0.6 (overall) at 2 years (*p* < 0.01). Active external rotation improved 52° at 6 months and 35° (total) at 2 years (*p* < 0.01) with an overall Mallet external rotation score improvement of 1.3 at 6 months (*p* < 0.01) and 0.52 (overall) at 2 years (*p* = 0.013). There was no statistically significant change in internal rotation (*p* = 0.37). We found no correlation between the child’s age or the severity of involvement at surgery and the end result.

**Conclusion:**

Proximal subscapularis release according to Carlioz is simple and effective in improving overall shoulder function in children with obstetric brachial plexus palsy, mainly abduction and external rotation. Improvement tends to reach a plateau around 6–12 months postoperatively.

## Introduction

Obstetric brachial plexus paralysis (OBPP) is the result of excessive stretching of the brachial plexus during difficult labor [1]. Although the majority of patients (60 %) recover spontaneously, the others retain functional impairment of variable severity [2].

The most commonly affected roots in OBPP are C5 and C6 [3]. If not properly treated, and even sometimes despite adequate treatment, C5–C6 injury may lead in some cases to adduction–internal rotation shoulder contracture [4]. Several surgical techniques are reported to improve shoulder abduction and external rotation and they include early reconstructive microsurgical repair of disrupted roots, open or arthroscopic subscapularis release, anterior open or arthroscopic glenohumeral capsulotomy, tendon transfer and/or humeral osteotomy [4–6].

Subscapularis contracture may prevent a functional rotator cuff from acting normally to produce optimal active shoulder abduction and external rotation, and releasing it may eliminate an obstacle to such shoulder function. Clinical evaluation of subscapularis contracture is based on a decrease in shoulder passive external rotation as assessed with the arm along the trunk and the elbow in 90° flexion.

In 1913, Fairbank treated OBPP by anterior capsulotomy and subscapularis tenotomy, but his procedures were followed by a high rate of complications [7]. In 1927, Sever amended Fairbank’s technique by subscapularis tenotomy without capsulotomy. The operated children had a significant loss of active and passive internal rotation [4]. In the early 70s, Carlioz and Brahimi described proximal subscapularis release in seven patients with OBPP and reported good results if surgery is performed at an optimal age (between 1 and 4 years), before any significant humeral head deformity appears [5]. Newman et al. reported a series of 13 children who underwent a proximal subscapularis release as the only treatment of OBPP with promising results [8].

Currently, there is no consensus concerning the technique that provides the best results for shoulder mobility and function. Similarly, there is a lack of evidence about clear surgical indications for OBPP. Moreover, subscapularis muscle release has not been widely discussed in the literature as an isolated treatment of OBPP. The purpose of this paper was to review the benefits and complications of proximal subscapularis release in children affected by this disorder and presenting with shoulder adduction–internal rotation contracture.

## Methods

A retrospective review was conducted of 64 consecutive children with OBPP who underwent an open proximal subscapularis release between 2001 and 2013 for shoulder adduction–internal rotation contracture. Exclusion criteria were a history of previous or associated surgery on the same shoulder, such as humeral osteotomy or tendon transfer. Fifty patients with complete records and a minimum 2-year follow-up were included. There were 25 girls and 25 boys with a mean age of 25 months (13–47) at surgery. The left side was involved in 20 cases and the right side in 30. C5–C6 roots were injured in 32 cases, C5–C6–C7 in 16 cases and the entire brachial plexus in two. All surgeries were performed by the senior author of this paper.

### Indications for surgery

Isolated proximal subscapularis release is part of the treatment algorithm set for every child diagnosed at birth with OBPP. It is intended for those children who do not succeed in achieving functional external rotation and abduction after the age of 1 year, despite a good rehabilitation program alone or following microsurgical brachial plexus repair between 3 and 6 months of life, and/or those who have more than 30° lack of passive external rotation compared to the opposite side. The optimal age for surgery ranges from 1 to 4 years and that is before major humeral head deformity appears. If improvement of active shoulder abduction and external rotation does not occur 1–2 years following surgery, then a Hoffer transfer (latissimus dorsi and teres major) may be indicated.

### Surgical technique

The technique is adapted from the original Carlioz procedure [4] with minor personal modifications, mainly concerning patient position and represented in *italics*.

**Fig. 1:**
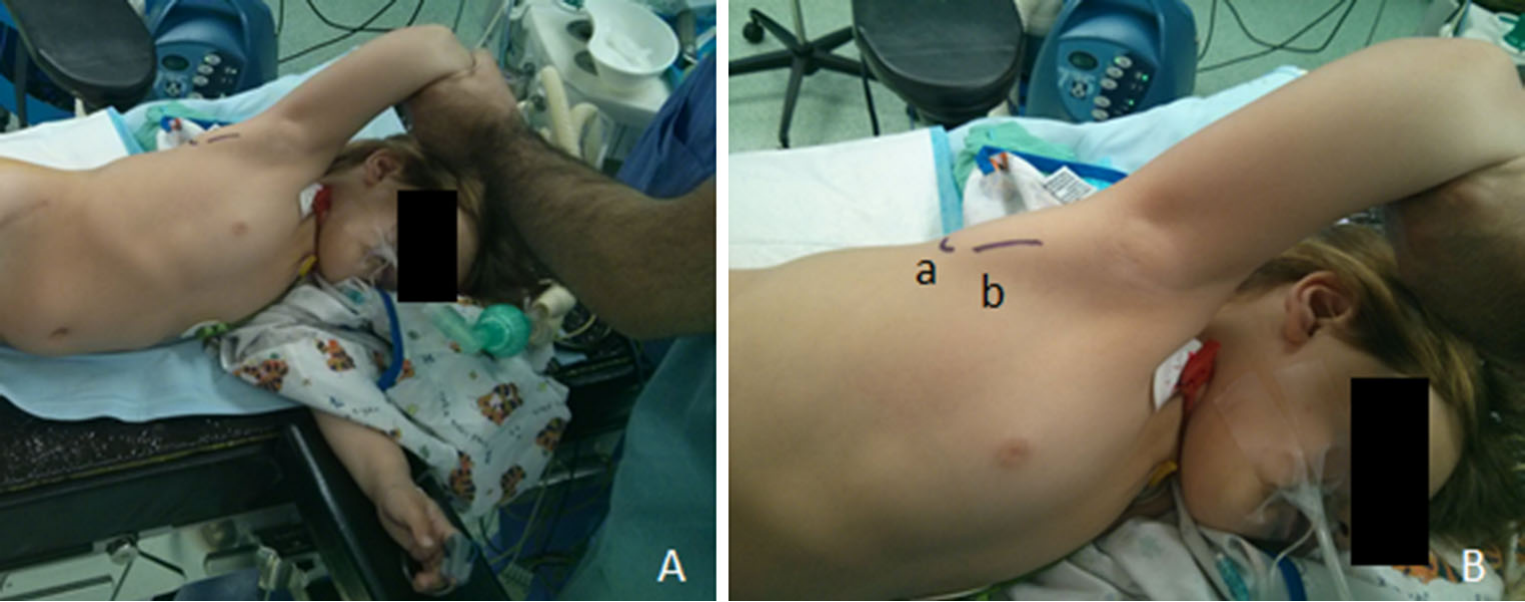
Patient lying in lateral decubitus with the side to be operated upwards. Notice the scapula bulging under the skin with forced abduction. *a* Tip of the scapula, *b* incision

**Fig. 2:**
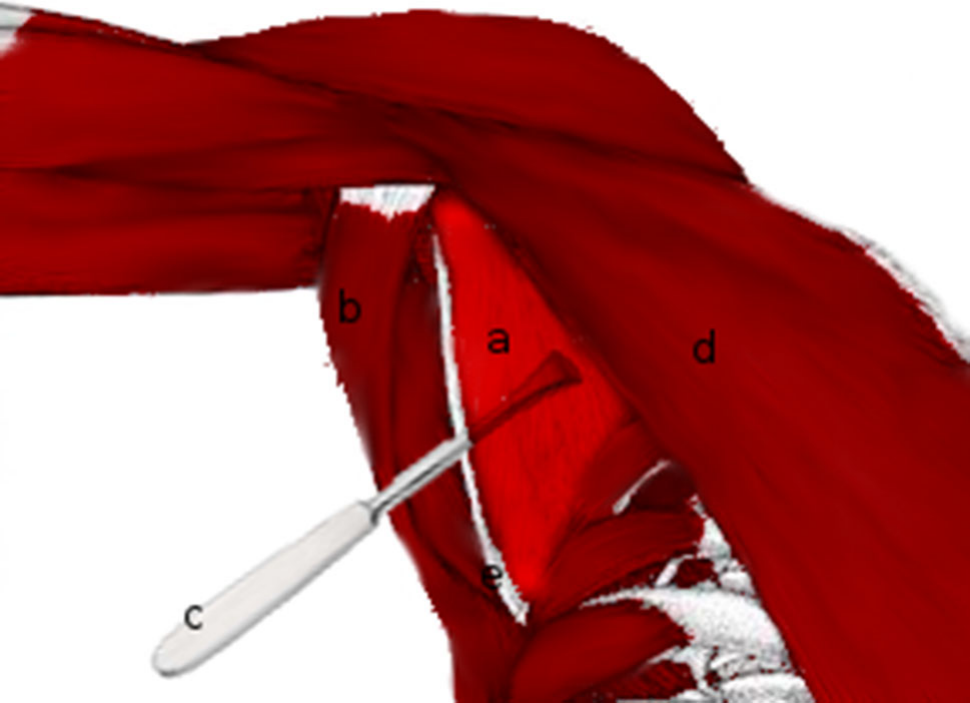
Extraperiosteal elevation of the subscapularis off the anterior aspect of the scapula. *a* Subscapularis, *b* latissimus dorsi, *c* periosteal elevator, *d* pectoralis major, *e* scapula

*The patient is placed in lateral decubitus with the side to be operated upwards. The upper limb is entirely draped in a routine fashion and positioned freely in extreme abduction so as to identify the prominence of the scapular’s tip and lateral border. The operating surgeon stands in front of the patient whereas the assistant stands opposite.* A 3-cm incision is performed along the lateral border of the scapula, starting 1 cm cephalad to its tip and extending cranially. The plane between latissimus dorsi and teres major is identified and dissected, the former retracted anteriorly and the latter posteriorly. The scapulothoracic fascia is then incised, thereby exposing the scapulothoracic space and the anterior surface of the subscapularis muscle. The latter is incised along its entire proximal most peripheral insertion on the scapula and stripped from it extraperiosteally, letting it freely slide distally on the anterior body of the scapula (Figs. 1, 2). The wound is checked for bleeders and a careful haemostasis is undertaken before it is closed in just two layers (subcutaneous and skin) in a regular manner without drainage. The upper limb is then placed in a shoulder spica in 90–100° of shoulder abduction, 90° of elbow flexion and 90° of external rotation for 5 weeks followed by cast bivalving, physical therapy and gradual weaning over a month. The child is then left without any external immobilization. Physical therapy is continued four times a week for 6 months, three times a week for 6 months, twice a week for 6 months and once a week until the end of growth.

### Data collection

**Fig. 3:**
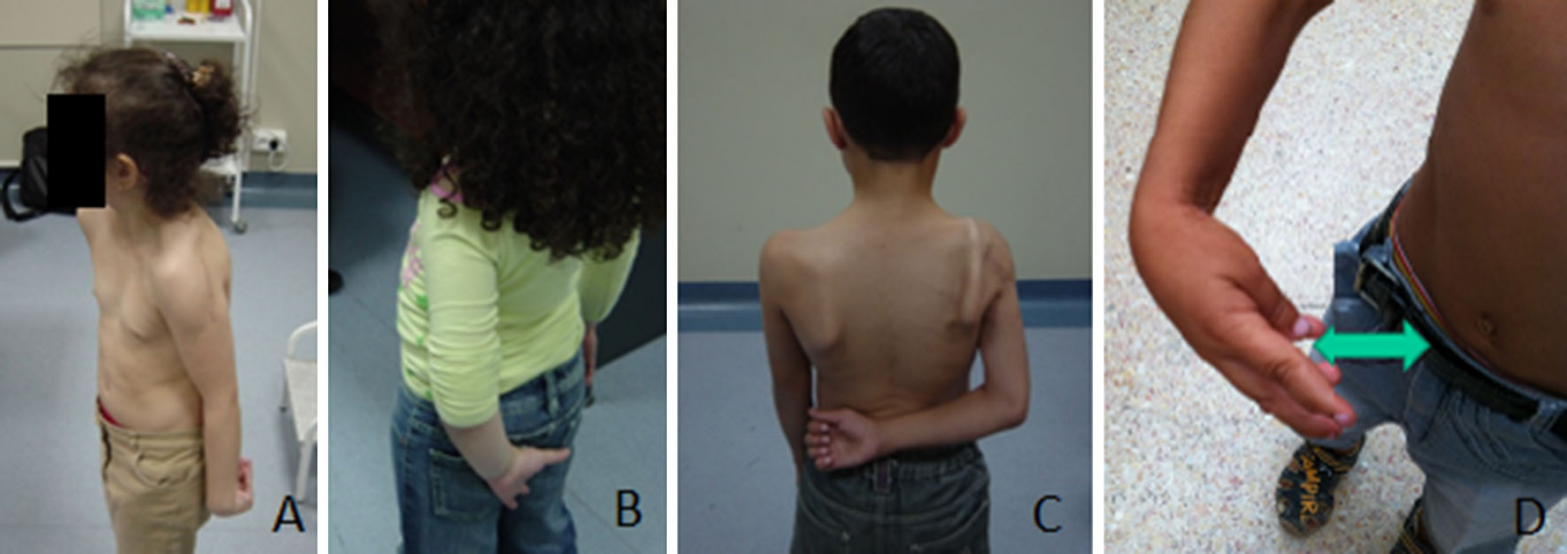
Internal rotation (IR) functional evaluation: **a** IR limited to the trochanter. **b** IR limited to the buttocks. **c** Normal IR. **d** Inability to button and unbutton the trousers

Patients were examined preoperatively and at regular intervals postoperatively and called back for the purpose of this study at an average follow-up of 4.2 years (2–8.2 years). Overall parent satisfaction as well as the impact of surgery on the child’s daily activity were recorded. Assessment of shoulder function improvement was based on documentation of postoperative modification of active abduction/forward flexion, active external and internal rotations separately as well as the improvement in Mallet score. In younger children, evaluation of active range of motion was based on stimulation of the upper limb in the desired direction. The disappearance of the trumpet sign in abduction was considered an improvement in active external rotation. Evaluation of internal rotation was modified from the one present in the Mallet score to make it more representative of the real impairment of daily function. (Fig. 3). The inability to button and unbutton the trousers due to lack of internal rotation accounted for unsuccessful and nonfunctional internal rotation.

### Data analysis

Immediate preoperative values were compared to 6-month and last follow-up values using a paired Student *t*-test with a 95 % confidence interval. Children younger than 3 years at surgery were compared to older ones and those with C5–C6 injury were compared to those with more extensive involvement. Statistical analysis was performed using SPSS 16.0 software (SPSS Inc., Chicago, IL, USA). *p* < 0.05 was considered statistically significant.

## Results

**Fig. 4:**
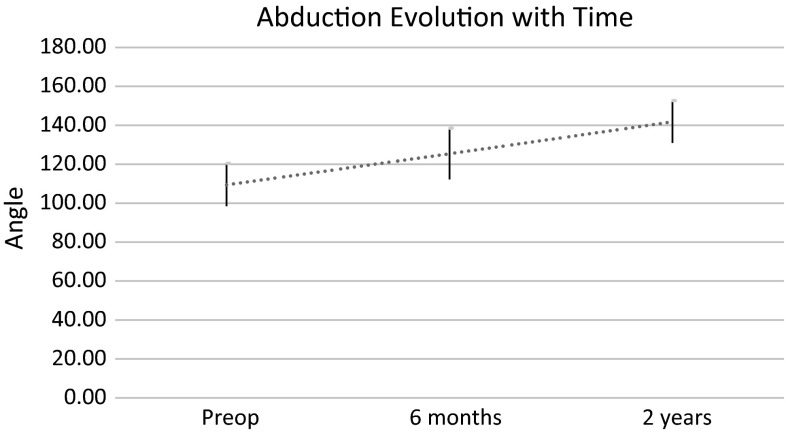
Evolution of abduction with time. *Vertical lines* 95 % confidence interval

**Fig. 5:**
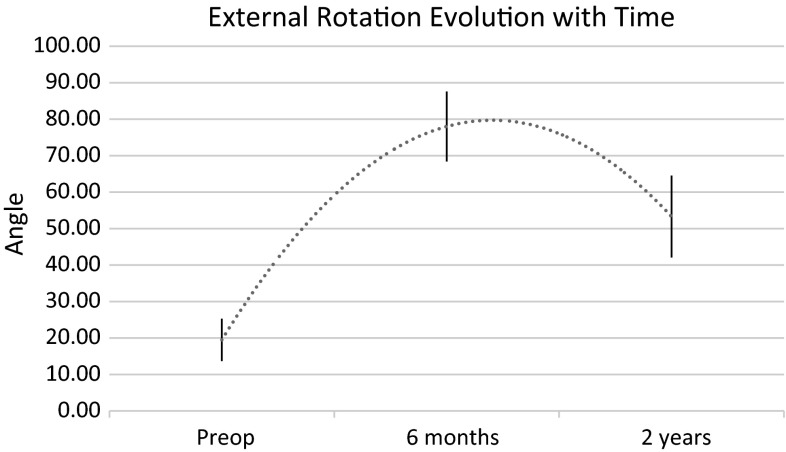
Evolution of external rotation with time. *Vertical lines* 95 % confidence interval

Active abduction improved 21° (15–45°) at 6 months and continued to do so up to last follow-up with an overall improvement of 31° (20–50°) (*p* < 0.01) (Fig. 4). The Mallet abduction score improved by 0.58 points (0.4–0.9) at 6 months and continued to do so over time reaching 0.6 points (0.45–0.95) at last follow-up (*p* < 0.01). Active external rotation improved 52° (40–60°) at 6 months but showed a gradual loss of improvement with time, reaching an overall improvement of 35° (25–45°) at last follow-up (*p* < 0.01) despite a sustained physical therapy program (Fig. 5). Similarly, the Mallet external rotation score improved by 1.3 points (1–1.7) at 6 months (*p* < 0.001) with an obvious gradual loss of improvement with time, reaching an overall positive modification of 0.52 points (0.45–0.8) at last follow-up (*p* = 0.013). We found no statistically significant change in active internal rotation (*p* = 0.37) but, in contrast, there was a slight tendency to worsening, without it reaching statistical significance.

We found no statistically significant correlation between the child’s age at surgery (<3 years vs >3 years) and the end result (Table 1), despite some tendency towards better results for children <3 years at surgery.

**Table 1:** Comparison between children operated before and after 3 years of age

	>3 years	<3 years	Difference	*p* value
Abduction (Mallet score)	0.42	0.63	−0.21	0.26
Abduction (angle)	27.14°	32.42°	−5.28°	0.34
External rotation (Mallet score)	0.25	0.56	−0.31	0.35
External rotation (angle)	35°	35.33°	−0.33°	0.49

In addition, there was no statistically significant difference in outcome between children with C5–C6 involvement and those more extensively affected (Table 2).

**Table 2:** Comparison between children with C5–C6 lesion only and others with more extended lesions

	All other roots	C5–C6	Difference	*p* value
Abduction (Mallet score)	0.41	0.60	−0.19	0.30
Abduction (angle)	22.91°	32.82°	−9.90	0.26
External rotation (Mallet score)	0.18	0.41	−0.22	0.32
External rotation (angle)	32.72°	31.76°	+0.96	0.46

Although the average shoulder abduction continued to improve between 6 months postoperatively and last follow-up, this was not statistically significant. However, the loss of gain in external rotation between the 6-month postoperative period and last follow-up was obvious and statistically significant (Table 3).

**Table 3:** Comparison between 6 months and last follow-up

	Difference between 6 months and last follow-up	*p* value
Mallet score for abduction	+0.125	0.16
Abduction angle	+8.2°	0.055
Mallet score for external rotation	−0.83	0.008*
External rotation angle	−17°	0.002*

* Statistically significant

We found no significant complications following surgery, mainly no wound dehiscence or infection, no hematoma and no nerve injury.

## Discussion

Reconstructive surgeries of the shoulder in children with OBPP have been performed since the early twentieth century to alleviate the sequels after nerve damage of the upper limb. Although it was one of the first practiced procedures, there are very few data in the literature describing results obtained from a subscapularis muscle release in OBPP [8]. There is currently a trend towards favoring tendon transfers as a first-choice surgical treatment in children with OBPP, probably due to reported insufficient improvement or a non-negligible rate of recurrence with an isolated subscapularis release [9]. Our study shows good short- and medium-term results following this procedure, mainly in abduction and external rotation as given by the improvement in Mallet score.

Our results are similar to those reported by Newman et al. [8] and Chen et al. [10] who showed a significant improvement in upper limb function based on the Mallet score following isolated subscapularis release at an average follow-up of 3.5 years and 1 year respectively. Although shoulder abduction continues to show some improvement 2 years after surgery, the greatest positive difference occurs during the first 6 months following surgery. Shoulder external rotation does not follow the same improvement pattern, since loss of postoperative improvement tends to occur over time despite a sustained rehabilitation program. We do not have any explanation for the discrepancy between abduction and external rotation improvement over time. Our findings are similar to those of Pichon and Carlioz [4], Carlioz and Brahimi [5] and Kirkos et al. [11]. The majority of their patients showed a progressive loss in shoulder mobility over time. They explained it by a possible secondary surrounding tissue contracture, degenerative changes in the glenohumeral joint later in adult life and finally by the fact that young teenagers tend to prefer to use the healthy limb. The lack of improvement or even in some cases worsening of internal rotation is the biggest unsolved problem of this procedure, sometimes interfering severely with activities of daily living. Sever reported a difficulty in internal rotation in almost all operated children 1 year after surgery [12]. Although regular and continuous physical therapy did not prevent the gradual loss of external rotation improvement after the first 6 postoperative months and did not improve internal rotation, the data reported in the current study does not allow us to recommend decreasing or discontinuing rehabilitation 6 months after surgery.

In the current study, we could not demonstrate the presence of a significant difference in functional outcome between children operated before the age of 3 years and those operated later in life. In addition, there does not seem to be any statistically significant difference in functional outcome between those children with C5–C6 and those with more severe involvement. This could be due to type II error.

This study had some limitations, such as the retrospective nature, the lack of a control group, and the fact that the operating surgeon is also the person who performed the follow-up evaluation.

## Conclusion

To our knowledge, this is one of the largest series reporting on the results of isolated subscapularis release in children with obstetric brachial plexus palsy. Isolated subscapularis release is an easy, efficient and safe procedure in young children with shoulder adduction–internal rotation contracture due to obstetric brachial plexus palsy. It improves abduction and external rotation but has no statistically significant influence on internal rotation.
